# Pterostilbene 4′-*β*-Glucoside Attenuates LPS-Induced Acute Lung Injury via Induction of Heme Oxygenase-1

**DOI:** 10.1155/2018/2747018

**Published:** 2018-10-23

**Authors:** Jeongmin Park, Yingqing Chen, Min Zheng, Jinhyun Ryu, Gyeong Jae Cho, Young-Joon Surh, Daisuke Sato, Hiroki Hamada, Stefan W. Ryter, Uh-Hyun Kim, Yeonsoo Joe, Hun Taeg Chung

**Affiliations:** ^1^Department of Biological Sciences, University of Ulsan, Ulsan 44610, Republic of Korea; ^2^Department of Neurology, Affiliated Hospital of YanBian University, YanJi 133000, China; ^3^Department of Anatomy, School of Medicine and Institute of Health Sciences, Gyeongsang National University, Jinju 52728, Republic of Korea; ^4^Tumor microenvironment Global Core Research Center and Research Institute of Pharmaceutical Sciences, College of Pharmacy, Seoul National University, Seoul 08733, Republic of Korea; ^5^Department of Life Science, Okayama University, Okayama 770-0005, Japan; ^6^Joan and Sanford I. Weill Department of Medicine, and Division of Pulmonary and Critical Care Medicine, Weill Cornell Medical Center, New York, NY 10065, USA; ^7^National Creative Research Laboratory for Ca^2+^ signaling Network, Chonbuk National University Medical School, Jeonju 54907, Republic of Korea

## Abstract

Heme oxygenase-1 (HO-1) can exert anti-inflammatory and antioxidant effects. Acute lung injury (ALI) is associated with increased inflammation and influx of proinflammatory cells and mediators in the airspaces and lung parenchyma. In this study, we demonstrate that pterostilbene 4′-*β*-glucoside (4-PG), the glycosylated form of the antioxidant pterostilbene (PTER), can protect against lipopolysaccharide- (LPS-) or *Pseudomonas aeruginosa- (P. aeruginosa*-) induced ALI when applied as a pretreatment or therapeutic post-treatment, via the induction of HO-1. To determine whether HO-1 mediates the antioxidant and anti-inflammatory effects of 4-PG, we subjected mice genetically deficient in *Hmox-1* to LPS-induced ALI and evaluated histological changes, HO-1 expression, and proinflammatory cytokine levels in bronchoalveolar lavage (BAL) fluid. 4-PG exhibited protective effects on LPS- or *P. aeruginosa-*induced ALI by ameliorating pathological changes in lung tissue and decreasing proinflammatory cytokines. In addition, HO-1 expression was significantly increased by 4-PG in cells and in mouse lung tissues. The glycosylated form of pterostilbene (4-PG) was more effective than PTER in inducing HO-1 expression. Genetic deletion of *Hmox-1* abolished the protective effects of 4-PG against LPS-induced inflammatory responses. Furthermore, we found that 4-PG decreased both intracellular ROS levels and mitochondrial (mt) ROS production in a manner dependent on HO-1. Pharmacological application of the HO-1 reaction product carbon monoxide (CO), but not biliverdin or iron, conferred protection in *Hmox-1*-deficient macrophages. Taken together, these results demonstrate that 4-PG can increase HO-1 expression, which plays a critical role in ameliorating intracellular and mitochondrial ROS production, as well as in downregulating inflammatory responses induced by LPS. Therefore, these findings strongly suggest that HO-1 mediates the antioxidant and anti-inflammatory effects of 4-PG.

## 1. Introduction

Sepsis-induced acute lung injury (ALI), an acute inflammatory disorder associated with alveolar-capillary barrier dysfunction, exhibits high rates of morbidity and mortality, despite modern clinical practices in critical care medicine [1–3]. Thus, there is an urgent need to develop effective treatments for ALI.

Several studies have indicated that dietary polyphenols including curcumin and resveratrol (3,5,4′-trihydroxystilbene) can exert protective effects in ALI by reducing the inflammatory response and enhancing antioxidant status [4, 5]. Resveratrol exhibits anti-inflammatory effects via suppression of the inflammatory cascade [6] and by reducing the release of inflammatory mediators including TNF-*α* and IL-8 [7]. Pterostilbene (PTER; 3,5-dimethoxy-4′-hydroxystilbene) is a phytoalexin and a natural dimethylated analog of resveratrol, which has been demonstrated to possess various resveratrol-like activities [8, 9]. PTER is a primary antioxidant component of blueberries [10]. Moreover, accumulating studies have shown that PTER can exert anti-inflammatory [11], anticarcinogenic [12], and antioxidant properties [10]. Glycosylation of bioactive compounds can enhance their intestinal absorption, as well as improve their bio- and pharmacological properties [13]. In our previous study, we demonstrated that treatment with the glycosylation product of PTER (pterostilbene 4′-*β*-glucoside, 4-PG) can attenuate dextran sulfate sodium-induced colitis in mice via upregulation of tristetraprolin (TTP) [14, 15]. However, whether 4-PG can exert a protective effect in endotoxin-induced ALI remains unclear. Furthermore, the underlying mechanism(s) by which 4-PG regulates inflammatory responses remains largely unknown.

Heme oxygenase (HO) catalyzes the first and rate-limiting step in the oxidative degradation of heme to generate biliverdin IX*α*, carbon monoxide, and iron. The stress-responsive protein, heme oxygenase-1 (HO-1), responds to induction by stimulants such as proinflammatory cytokines, heat shock, heavy metals, and oxidants [16, 17]. The antioxidant and anti-inflammatory effects of PTER have been shown to require activation of HO-1 [11, 18]. For example, recent evidence indicates that PTER treatment can alleviate cerebral ischemia/reperfusion (I/R) injury via the activation of HO-1-dependent signaling [19]. However, whether HO-1 mediates the anti-inflammatory effects of 4-PG remains unclear.

In the present study, we demonstrate that 4-PG can attenuate oxidative stress and inflammatory responses in LPS-activated murine RAW 264.7 macrophages and in mouse models of LPS- or *P. aeruginosa*-induced ALI, via the enhancement of HO-1. Additionally, after LPS-challenge, 4-PG displayed greater benefit with respect to the activation of HO-1 and anti-inflammatory responses, than that of its parent compound PTER. Thus, 4-PG may have potential therapeutic value for the treatment of ALI.

## 2. Methods

### 2.1. Reagents and Chemicals

Pterostilbene 4′-*β*-glucoside (4-PG) was donated from Okayama University of Science, Dept. of Life Science. Pterostilbene (PETR) was purchased from Tokyo Chemical Industry (TCI), Japan. 4-PG and PTER were dissolved in DMSO, as described previously [14]. Lipopolysaccharide (LPS), carbon monoxide-releasing molecule- (CORM-) 2, bilirubin, and ammonium iron(II) sulfate hexahydrate were purchased from Sigma-Aldrich, (St. Louis, MO, USA). Antibody against HO-1 was purchased from Enzo Life Sciences (Farmingdale, NY, USA), and antibody against *β*-actin was purchased from Cell Signaling (Danvers, MA, USA). siRNA against HO-1 was from Santa Cruz Biotechnology (Santa Cruz, CA). Zinc protoporphyrin IX (ZnPP) was from Frontier Scientific (Logan, UT, USA).

### 2.2. Cell Culture

Mouse macrophage RAW 264.7 cells (KCLB, Seoul, Korea) were cultured in DMEM containing 10% FBS and 1% penicillin-streptomycin solution, at 37°C in humidified incubators containing an atmosphere of 5% CO_2_. RAW 264.7 cells were pretreated with 4-PG (10 *μ*M) and then stimulated with LPS (100 ng/ml). For silencing the HO-1 gene, RAW 264.7 cells were transfected with siRNA against mouse HO-1 using Lipofectamine 2000 (Invitrogen, CA) according to the manufacturer's protocol. Human macrophage U937 cells and human lung epithelial A549 cells (KCLB, Seoul, Korea) were cultured in RPMI 1640 medium (Gibco, Grand Island, NY, USA), containing 10% FBS and 1% penicillin-streptomycin, at 37°C in humidified incubators containing an atmosphere of 5% CO_2_. Bone marrow-derived macrophages (BMDM) from *Hmox-1^+/+^* and *Hmox-1^−/−^* mice were isolated as previously described [14]. Peripheral blood mononuclear cells (PBMC) were isolated from the blood by centrifugation on Ficoll-Paque Plus density gradient medium (GE Healthcare, Little Chalfont, UK). The PBMC layer was collected and centrifuged at 2000 rpm for 20 minutes to remove the remaining Ficoll solution. PBMC were collected in DMEM and plated.

### 2.3. Animals

BALB/c *Hmox-1^−/−^* mice were obtained by Dr. Mark A. Perrella (Brigham and Women's Hospital, Boston, MA). Mice were bred in the animal facility at the University of Ulsan and were born and housed in the same room under specific pathogen-free conditions at 18–24°C and 40–70% humidity, with a 12 h light-dark cycle. All mice were handled in accordance with guideline of the Institutional Animal Care and Use Committee (IACUC) of the University of Ulsan.

### 2.4. LPS-Induced ALI and 4-PG Treatment

*Hmox-1^+/+^* (*n* = 30) and *Hmox-1^−/−^* (*n* = 30) mice (8–10 weeks old, 20–25 g) were randomly divided into six groups (*n* = 5 in each group): control and LPS-, LPS + 4-PG-, LPS + PTER-, 4-PG-, and PTER-treated groups. For induction of ALI, LPS (2.5 mg/kg) was applied once by intranasal administration in mice and then mice were sacrificed after 24 h. To evaluate the preventive effects of 4-PG and PTER, 4-PG (10 mg/kg, i.p.) or PTER (10 mg/kg, i.p.) was injected for 4 days prior to LPS challenge. After 24 h, bronchoalveolar lavage (BAL) fluid was collected by flushing the lung with 1 ml of PBS. To assess the therapeutic effects of 4-PG and PTER on LPS-induced ALI, 8-week-old C57BL/6 mice were challenged with LPS (2.5 mg/kg) by intranasal administration. After 5 h, mice were post-treated with 4-PG (10 mg/kg, i.p.) or PTER (10 mg/kg, i.p.) for an additional 19 h in the presence or absence of LPS stimulation. After 24 h, bronchoalveolar lavage (BAL) fluid was collected by flushing the lung with 1 ml of 1x PBS (Gibco, Grand Island, NY, USA), and the number of cells in BAL fluid was measured using a hemocytometer. Lung tissues were harvested to observe the pathological changes by H&E staining and to measure the expression of proinflammatory cytokines as well as chemokines by RT-PCR, Western blot, and ELISA.

### 2.5. *Pseudomonas aeruginosa*-Induced ALI and 4-PG Treatment

To estimate the therapeutic effects of 4-PG on *Pseudomonas aeruginosa-*induced ALI, 8-week-old C57BL/6 mice were randomly dived into five groups: Control, *P. aeruginosa*, *P. aeruginosa* + 4-PG 6 h, *P. aeruginosa* + 4-PG 12 h, and *P. aeruginosa* + 4-PG 18 h. To construct the *P. aeruginosa*-induced pneumonia mouse model, mice were treated with *P. aeruginosa* (1 × 10^7^ CFU in 20 *μ*l PBS per mouse) through intranasal instillation for 24 h. After administration of *P. aeruginosa*, mice were post-treated with 4-PG at 6, 12, and 18 h (10 mg/kg, i.p.), respectively. After 24 h, mice were sacrificed and bronchoalveolar lavage (BAL) fluid was collected by flushing the lung with 1 ml of PBS, and the number of cells in BAL fluid was measured by using a hemocytometer and proinflammatory cytokines by ELISA. Lung tissues were harvested to observe the neutrophil infiltration by MPO measurement.

### 2.6. Lung Wet-to-Dry Weight Ratio

To measure the lung edema in *Pseudomonas aeruginosa* pneumonia, whole-lung tissues were collected and surface blood was removed. Then, the weights of samples were recorded as the wet weight, and tissues were dried for 72 h at 65°C and recorded as the dry weight. Pulmonary edema was expressed as a wet/dry weight ratio for each individual mouse lung.

### 2.7. Myeloperoxidase (MPO) Assay

To measure neutrophil infiltration into lung tissues, MPO enzyme activity in lung tissues was measured using the Mouse Myeloperoxidase DuoSet kit (R&D Systems, Minneapolis, MN).

### 2.8. MTT Assay

To perform MTT assay, RAW 264.7 cells were grown in a 96-well plate. After reaching 70% confluence, the cells were treated with 4-PG at the indicated concentrations (5, 10, 15, 20, and 40 *μ*M) or PTER at the indicated concentrations (10, 15, 20, and 40 *μ*M) for 8 h. The cells were incubated with MTT (thiazolyl blue tetrazolium bromide, Sigma-Aldrich) for 4 h. To quantify cell viability, MTT formazan was eluted with 100% isopropanol and the optical density of samples was read at 570 nm on a spectrophotometer.

### 2.9. RNA Isolation and Reverse Transcription-Polymerase Chain Reaction

Total RNA was isolated from RAW 264.7 cells and lung tissues using TRIzol reagent (Invitrogen). 2 *μ*g of total RNA was used to synthesize cDNA by using M-MLV reverse transcriptase (Promega). The synthesized cDNA was subject to PCR-based amplification. The following primers were mouse GAPDH (f-aggccggtgctgagtatgtc, r-tgcctgcttcaccttct), mouse TNF-*α* (f-agcccacgtcgtagcaaaccaccaa, r-acacccattcccttcacagagcaat), mouse IL-6 (f-gtggaaatgagaaaagagttgt, r-cctcttggttgaagatatgaat), mouse IL-1*β* (f-ctgtgtctttcccgtggacc, r-cagctcatatgggtccgaca), mouse CXCL1 (f-gctgggattcacctcaagaa, r-gcacttcttttcgcacaaca), mouse CXCL2 (f-cagactccagccacacttca, r-aggcacatcaggtacgatcc), mouse *Hmox-1* (f-tcccagacaccgctcctccag, r-ggatttggggctggtttc) and mouse 18S (f-cagtgaaactgcgaatggct, r-tgccttccttggatgtggta), human GAPDH (f-ccacccatggcaaattccatggca, r-tctagacggcaggtcaggtccacc), human HO-1 (f-cttcgcccctgtctacttcc, r-gtccttggtgtcatgggtca), human IL-6 (f-ctctatggagaactaaaagt, r-actgcatagccactttccat), and human TNF-*α* (f-gagcactgaaagcatgatccg, r-aaagtagacctgcccagactcgg). To perform real-time quantitative PCR (RT-qPCR), the synthesized cDNA was amplified with SYBR Green qPCR Master Mix (2x, USB Production; Affymetrix) on an ABI 7500 Fast Real-Time PCR System (Applied Biosystems, Carlsbad, CA). The following RT-qPCR primers were mouse GAPDH (f-cggcctcaccccatttg, r-gggaagcccatcaccatct), mouse TRX1 (f-atggtgaagatcgagagc, r-ggcatattcagtaatagaggc), mouse NQO1 (f-agctggaagctgcagacctg, r-cctttcagaatggctggca), and mouse GCLC (f-atctgcaaaggcggcaac, r-actcctctgcagctggctc).

### 2.10. Dual Luciferase Assay

For dual luciferase assay, RAW 264.7 cells were grown in a 96-well plate, and then cells were cotransduced with a pCignal Lenti-ARE reporter (Qiagen, Hilden, Germany) and pCignal Lenti-TK-Renilla (Qiagen, Hilden, Germany). After 72 h, cells were treated with 10 *μ*M 4-PG for 8 h. Treated cells were lysed with passive lysis buffer (Promega, Fitchburg, WI, USA) and mixed with luciferase assay reagents (Promega). The chemiluminescent signal was detected using a SpectraMax L Microplate reader (Molecular Devices, Sunnyvale, CA). Firefly luciferase was normalized to Renilla luciferase in each sample.

### 2.11. Western Blot

Total proteins extracted from harvested tissues and cells were prepared in mammalian lysis buffer containing phosphatase and protease inhibitors, and the protein concentration was determined using the BCA protein assay kit (Pierce Biotechnology, Rockford, IL, USA). The protein was fractionated on polyacrylamide-SDS gels and transferred to polyvinylidene difluoride membranes. The membrane was blocked with 5% nonfat milk in phosphate-buffered saline Tween 20 (PBS-T) and then incubated with a primary antibody against HO-1 (1 : 2000 *v*/*v* in PBS-T, Enzo Life Sciences, USA) or *β*-actin (1 : 2500 *v*/*v* in PBS-T, Cell Signaling Technology, MA) followed by incubation with a secondary antibody. Antibody binding was visualized with an ECL chemiluminescence system (GE Healthcare Bio-Sciences, Little Chalfont, UK).

### 2.12. Enzyme-Linked Immunosorbent Assays (ELISA)

Cytokines were analyzed in cell culture supernatants or BAL fluid recovered from mice. The concentrations of IL-6 and TNF-*α* were measured by using BioLegend MAX™ ELISA kits according to the manufacturer's instructions (BioLegend, San Diego, USA).

### 2.13. Intracellular ROS Measurement

RAW 264.7 cells were pretreated with 4-PG (10 *μ*M) for 6 h or with ZnPP (10 *μ*M) for 30 min, respectively, followed by stimulation with LPS (100 ng/ml) for an additional 4 h. Then, cultured medium was replaced with PBS containing 5 *μ*M CM-H_2_DCFDA (C6827, Invitrogen, CA) for 45 min at 37°C. Intracellular ROS was assessed using a flow cytometer with a fluorescence-activated cell sorter (FACSCanto II), and data was analyzed by FlowJo V10 software (Tree Star Inc., San Carlos, CA). To evaluate ROS by confocal microscopy, RAW 264.7 cells were grown in a confocal 4-well chamber. After reaching 70% confluence, the cells were transfected with siHO-1 RNA for 36 h and then incubated with 10 *μ*M 4-PG for 6 h and cultured in medium with 100 ng·ml^−1^ LPS for an additional 4 h. Cells were stained with 10 *μ*M CM-H_2_DCFDA (Invitrogen, C6827) for 1 h and then washed four times with PBS. Images were obtained using an Olympus FV1200 confocal microscope (Olympus, Tokyo, Japan).

### 2.14. Mitochondrial ROS Measurement

To perform mtROS measurement by FACS, RAW 264.7 cells were grown in 6-well plates. After reaching 80% confluence, the cells were incubated to 10 *μ*M 4-PG for 6 h and cultured in medium with 100 ng/ml LPS for an additional 4 h in the absence or presence of ZnPP (10 *μ*M). *Hmox-1^+/+^* and *Hmox-1^−/−^* BMDM were pretreated with 4-PG (10 *μ*M) or MitoTEMPO (100 *μ*M) (Billerica, MA, USA) for 6 h or 30 min, respectively, then cells were stimulated with LPS (100 ng/ml) for another 4 h. A549 cells were pretreated with 4-PG (10 *μ*M) for 4 h or with MitoTEMPO (100 *μ*M) for 30 min, respectively, and then stimulated with LPS (10 *μ*g/ml) for an additional 6 h. After treatment, cells were stained with MitoSOX Red (5 *μ*M) for 30 min and then washed four times with PBS. mtROS was assessed by using flow cytometry with a fluorescence-activated cell sorter (FACSCanto II), and data was analyzed by FlowJo V10 software (Tree Star Inc., San Carlos, CA).

### 2.15. Histology

Lung tissues were fixed in formalin solution, neutral buffered at 10%, and then embedded in paraffin, cut into 5 *μ*m thick sections, and stained with hematoxylin and eosin. Severity of lung injury was evaluated and scored in a blinded manner based on four aspects: congestion of alveolar-capillary membrane, hemorrhage, infiltration or aggregation of neutrophils in the air space or the vessel wall, and thickness of the alveolar wall/hyaline membrane formation. Each of the four components was assessed ranging from 0 to 4, whereby a higher number is more severe. All of scores were added to generate a clinical score that categorized from 0 to 16 [31].

### 2.16. Statistical Analysis

For statistical comparisons, all values were expressed as mean ± SD. Statistical differences between samples were assessed by ANOVA with post hoc Tukey's honestly significant difference (HSD) test. Moreover, for statistical differences between groups in *Hmox-1*^+/+^ and *Hmox-1^−/−^* genotypes, as well as in scRNA and siHO-1-transfected cells, data were assessed by two-way ANOVA with Bonferroni post-tests. Data were analyzed and presented with GraphPad Prism software version 5.03 (San Diego, CA). Probability values of *p* ≤ 0.05 were considered to represent a statistically significant change.

## 3. Results

### 3.1. 4-PG Prevents LPS-Induced Acute Lung Injury and Upregulates HO-1 Expression

**Figure 1:**
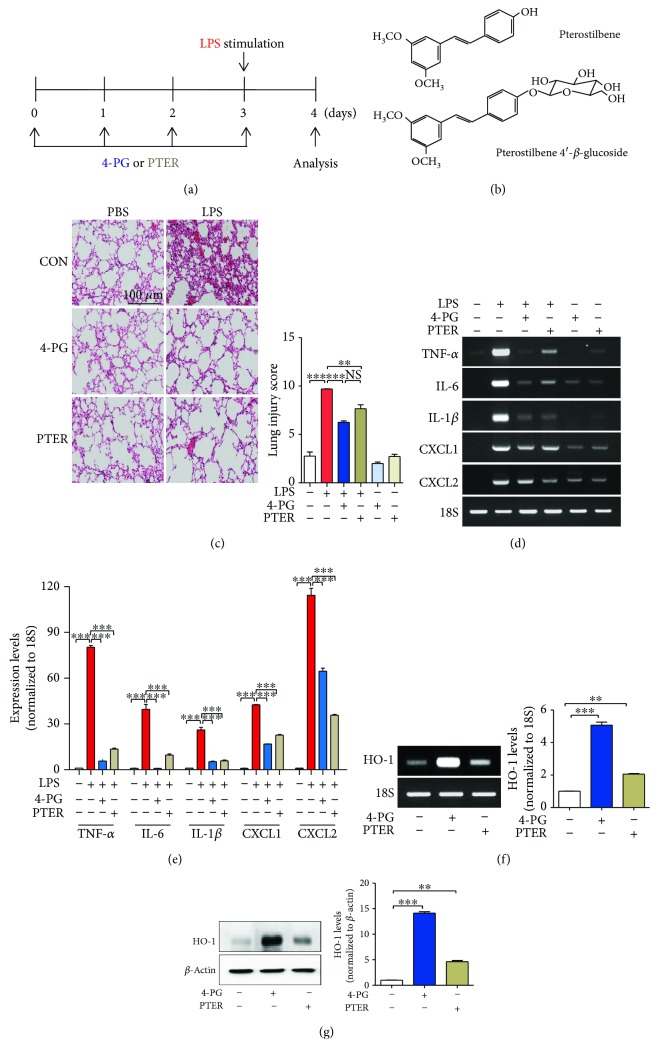
4-PG prevents LPS-induced acute lung injury and upregulates HO-1 expression. (a) The scheme depicts the experimental protocol used to assess the protective effect of pterostilbene 4′-glucoside (4-PG) and pterostilbene (PTER) on LPS-induced ALI. 10-week-old mice were injected with 4-PG (10 mg/kg, i.p.) and PTER (10 mg/kg, i.p.) for 4 days prior to intranasal administration of LPS (2.5 mg/kg) for 24 h. (b) Chemical structures of 4-PG and PTER. (c) Lung sections were stained with hematoxylin and eosin (H&E) for morphological evaluation, and the representative lung sections of each group are shown. Scale bar = 100 *μ*m. (left). Quantitative analysis of histologic lung section by lung injury score for six experimental groups. The score generates the average of two independent investigators (right). (d, e) The mRNA expression of proinflammatory cytokines and chemokines (TNF-*α*, IL-6, IL-1*β*, CXCL1, and CXCL2) in lung tissues was detected by RT-PCR. Furthermore, mRNA and protein levels of HO-1 were assessed by RT-PCR (f, left: HO-1mRNA levels, right: quantification of the relative band density) and Western blotting (g, left: HO-1 protein levels, right: quantification of the relative band density) from lung tissues, respectively. 18S and *β*-actin were used as internal controls. Data were expressed as mean ± SD (*n* = 5 per group); ^∗∗^*p* < 0.01 and ^∗∗∗^*p* < 0.001. Comparisons were made by one-way ANOVA with Turkey post hoc tests.

To investigate the anti-inflammatory effects of 4-PG and PTER, we first established an LPS-induced ALI model in mice. 10-week-old mice were injected with 4-PG (10 mg/kg, i.p.) or PTER (10 mg/kg, i.p.) for 4 days prior to intranasal administration of LPS (2.5 mg/kg) for 24 h (see Figure 1(a) for experimental design and Figure 1(b) for compound structures). Subsequently, we analyzed the protective effect of 4-PG on LPS-induced ALI. Lung histology was determined by hematoxylin and eosin (H&E) staining of lung sections. Administration of LPS caused lung injury in mice, reflected by thickening of the alveolar septum and inflammatory cell infiltration (Figure 1(c)). Treatment with 4-PG or PTER substantially reduced LPS-induced lung injury (Figure 1(c)). Previous studies have demonstrated that endotoxin-induced proinflammatory cytokines such as TNF-*α*, IL-1*β*, and IL-6 can contribute to the development of ALI [20–22]. Moreover, activation of circulating neutrophils and their transmigration into the alveolar airspace are associated with development of ALI. Neutrophil sequestration into the alveolar compartment is regulated by CXC chemokine receptor (CXCR2) and its ligands (CXC chemokine ligand CXCL1–8) [23]. Thus, we next assessed the mRNA expression of proinflammatory cytokines and chemokines (TNF-*α*, IL-6, IL-1*β*, CXCL1, and CXCL2) in lung tissue. As expected, 4-PG and PTER significantly attenuated the expression of inflammation-associated cytokines and chemokines (Figures 1(d) and 1(e)). Additionally, 4-PG was more effective than PTER at decreasing the TNF-*α*, IL-6, and CXCL1 mRNA expression. Subsequently, we evaluated HO-1 levels in lung tissues and found that 4-PG significantly induced both mRNA and protein levels of HO-1. Interestingly, compared with the administration of PTER, 4-PG was more potent with regard to enhancing HO-1 expression (Figures 1(f) and 1(g)).

### 3.2. 4-PG Exhibits Therapeutic Effects in LPS- or *P. aeruginosa*-Induced Acute Lung Injury

**Figure 2:**
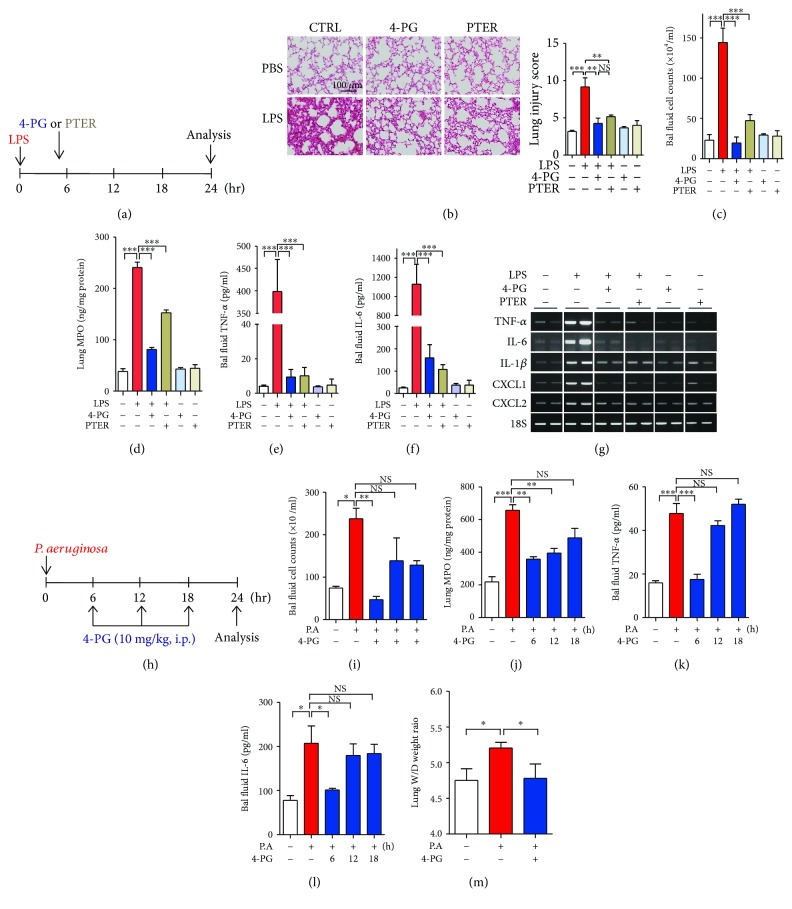
4-PG and PTER exhibit therapeutic effects in LPS- or *P. aeruginosa*-induced acute lung injury. (a–g) C57BL/6 mice were challenged with LPS (2.5 mg/kg) by intranasal exposure for 24 h. 4-PG (10 mg/kg per day, i.p.) and PTER (10 mg/kg per day, i.p.) were administered 5 h after LPS challenge. (b) Representative lung histology was shown by hematoxylin and eosin- (H&E-) stained lung sections from six experimental groups (left). Quantitative analysis of histologic lung section by lung injury score for six experimental groups. The score generates the average of two independent investigators (right). (c and d) BAL fluid and lung tissues were harvested after treatment with LPS for 24 h and then (c) analyzed for total cells in BAL fluid. (d) Neutrophil infiltration into lung tissues was measured by activity of myeloperoxidase (MPO) in lung tissues. (e and f) The secreted levels of TNF-*α* and IL-6 in BAL fluid were analyzed by ELISA, respectively. (g) The mRNA levels of TNF-*α*, IL-6, IL-1*β*, CXCL1, and CXCL2 in lung tissues were detected by RT-PCR. (h–m) C57BL/6 mice were instilled *P. aeruginosa* (1 × 10^7^ CFU/mouse) by intranasal exposure for 24 h. After instillation of *P. aeruginosa*, mice were post-treated with 4-PG (10 mg/kg, i.p.) at 6, 12, and 18 h, respectively. After 24 h, BAL fluid and lung tissues were collected and then (i) analyzed for total cells in BAL fluid. (j) Neutrophil infiltration into lung tissues was analyzed by MPO in lung tissues. (k and l) The levels of TNF-*α* and IL-6 in BAL fluid were analyzed by ELISA, respectively. (m) The wet-to-dry weight ratio of whole lungs was determined on 24 h after the stimulation of *P. aeruginosa*. Data were expressed as means ± SD, *n* = 3. ^∗^*p* < 0.05, ^∗∗^*p* < 0.01, and ^∗∗∗^*p* < 0.001.

Next, to evaluate the therapeutic effects of 4-PG on LPS-induced ALI, mice were challenged with LPS via intranasal administration for 5 h and then treated with 4-PG or PTER for another 19 h (Figure 2(a)). To investigate the effects of 4-PG or PTER post-treatment on LPS-induced pulmonary inflammation, bronchoalveolar lavage (BAL) fluid and lung tissues were analyzed at 24 h after LPS administration. In BAL fluid, the number of total cells was increased after LPS challenge. However, this increase was attenuated by 4-PG or PTER post-treatment (Figure 2(b)). The activity of myeloperoxidase (MPO), a major component of neutrophil cytoplasmic granules, can serve as a marker of infiltrated neutrophils in lung tissues. MPO activity was therefore analyzed to determine the extent of neutrophil infiltration in lung tissues. 4-PG or PTER post-treatment prevented LPS-induced MPO activity (Figure 2(c)). Lung histology revealed lung injury in the LPS-treated group. However, treatment with 4-PG or PTER after LPS administration effectively reduced the MPO content in lung tissue (Figure 2(d)). We next detected the levels of proinflammatory cytokines, TNF-*α* and IL-6, in BAL fluid. The protein levels of TNF-*α* and IL-6 significantly increased at 24 h of LPS administration, whereas post-treatment with 4-PG or PTER substantially reduced proinflammatory cytokines (Figures 2(e) and 2(f)). Additionally, we analyzed the mRNA levels of proinflammatory cytokines, TNF-*α*, IL-6, and IL-1*β*, and chemokines, CXCL1 and CXCL2, in lung tissues. 4-PG or PTER post-treatment significantly reduced the increase of proinflammatory cytokines and chemokines during LPS-induced pulmonary inflammation (Figure 2(g)). *P. aeruginosa* is a bacterial pathogen causing acute and chronic pulmonary infection in immunocompromised people [24]. Human pneumonia is modeled with experimental infections of animals, most frequently mice. Mouse models are leading to important discoveries relevant to pneumonia, and several approaches to establishing pneumonia in mice have been developed [25]. Based on these reports, to evaluate whether 4-PG protects against *P. aeruginosa*-induced pneumonia, 8-week-old mice received *P. aeruginosa* (1 × 10^7^ CFU) by intranasal instillation for 24 hours, and these mice were post-treated with 4-PG (10 mg/kg, i.p.) at 6, 12, and 18 hours, respectively, after the administration of *P. aeruginosa* (Figure 2(h)). We first examined the cell count in BAL fluid and observed that compared with the 24-hour infection of *P. aeruginosa* alone, the post-treatment of 4-PG at 6 hours dramatically reduced the cell count, while the mice post-treated with 4-PG at a late time, 12 and 18 h, showed no significant differences in BAL fluid cell number (Figure 2(i)). Subsequently, we found that the increased activity of MPO in the *P. aeruginosa*-induced pneumonia group was significantly reduced after the post-treatment of 4-PG groups at 6 and 12 h, while the post-treatment group at 18 h showed no significant changes in MPO concentration (Figure 2(j)). Furthermore, we detected proinflammatory cytokines, TNF-*α* and IL-6, and similar results were obtained that the secreted protein levels of TNF-*α* and IL-6 in BAL fluid were dramatically decreased under the treatment of 4-PG at 6 h after the stimulation of *P. aeruginosa*. However, the post-treatment at 12 and 18 h showed no significant differences compared with the group treated with *P. aeruginosa* alone (Figures 2(k) and 2(l)). Then, we estimated the wet-to-dry weight ratio of the lung tissues and observed that *P. aeruginosa*-infected mice elicited a significant rise in the wet-to-dry ratio when compared with PBS-treated mice, while the mice post-treated with 4-PG at 6 h significantly decreased the wet-to-dry lung weight ratio. In comparison, no significant differences were observed at late time, 12 and 18 h, post-treatment (Figure 2(m)). These data strongly suggest that 4-PG and PTER can also exhibit therapeutic effects in LPS- or *P. aeruginosa*- induced ALI. Additionally, according to the time course experiments, we found that the feasible therapeutic application of 4-PG in ALI is early-time (6-hour) but not late time (12- and 18-hour) administration. These data suggest that 4-PG or PTER post-treatment can also exhibit therapeutic effects in LPS- or *P. aeruginosa*- induced ALI.

### 3.3. 4-PG Induces HO-1 mRNA and Protein Expression in Macrophages and Lung Epithelial Cells

**Figure 3:**
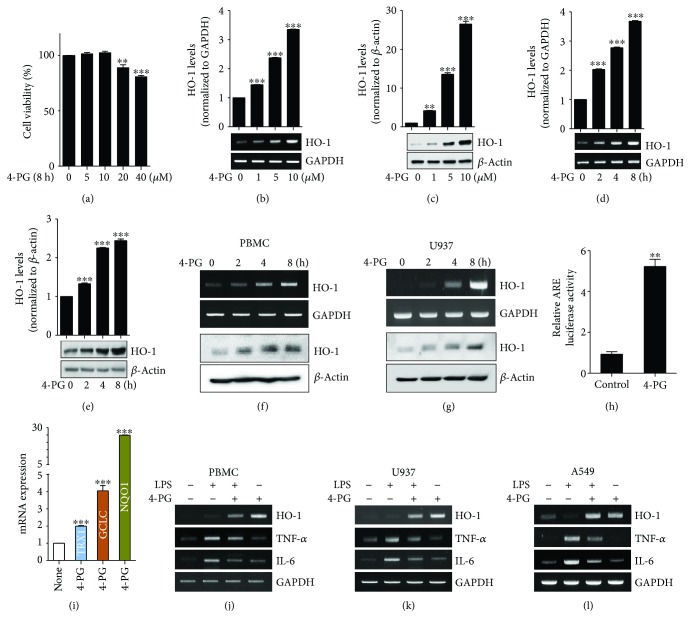
4-PG induces HO-1 mRNA and protein expression in macrophages and lung epithelial cells. (a) RAW 264.7 cells were treated with 4-PG at various concentrations (0, 5, 10, 20, and 40 *μ*M) for 8 h, and cell viability was determined by MTT assay. To evaluate the beneficial effect of 4-PG on HO-1 induction, cells were treated with 4-PG (0, 1, 5, and 10 *μ*M) at the indicated concentrations for 8 h. The mRNA and protein levels of HO-1 were measured by RT-PCR (b) and Western blotting (c). RAW 264.7 cells were treated with 4-PG (10 *μ*M) at the indicated time points (0, 2, 4, and 8 h). The mRNA and protein levels of HO-1 were determined by RT-PCR (d) and Western blotting (e). (f and g) PBMC and U937 cells were treated with 4-PG at the indicated concentrations (0, 1, 5, and 10 *μ*M) for 8 h. The mRNA and protein levels of HO-1 were determined by RT-PCR (top) and Western blotting (bottom). GAPDH and *β*-actin were used as internal controls. (h) RAW 264.7 cells were cotransduced with a pCignal Lenti-ARE reporter and pCignal Lenti-TK-Renilla. After treatment with 4-PG, luciferase activity was analyzed. The expression levels obtained from pCignal Lenti-ARE reporter-transduced cells without 4-PG treatment were normalized to 1. (i) RAW 264.7 cells were treated with 4-PG (10 *μ*M) for 8 h. Several antioxidative genes including TRX1, GCLC, and NQO1 were measured by RT-qPCR. (j and k) PBMC and U937 cells were pretreated with 4-PG (10 *μ*M) for 6 h followed by the stimulation of LPS (100 ng/ml) for another 4 h. (l) A549 cells pretreated with 4-PG (10 *μ*M) for 4 h and then stimulated with LPS (10 *μ*g/ml) for 6 h. The mRNA levels of HO-1, TNF-*α*, and IL-6 were determined by RT-PCR. Data were expressed as mean ± SD (*n* = 5 determined in five independent experiments). One-way ANOVA with Turkey post hoc tests were performed; ^∗^*p* < 0.05, ^∗∗^*p* < 0.01, and ^∗∗∗^*p* < 0.001 vs. the vehicle control group.

To define whether 4-PG enhances the expression of HO-1 *in vitro*, we first assessed the cytotoxic effect of 4-PG on murine macrophage RAW 264.7 cells. Cells were treated with 4-PG at various concentrations (0, 5, 10, 20, and 40 *μ*M) to detect cell viability using the MTT assay. As shown in Figure 3(a), no cellular toxicity was observed with the treatment of 4-PG at doses of 5 and 10 *μ*M for 8 h, while significant cytotoxicity was detected at 20 *μ*M. We therefore used 4-PG at a maximum concentration of 10 *μ*M in subsequent experiments. RAW 264.7 cells were treated with 4-PG at the indicated concentrations (0, 1, 5, and 10 *μ*M) and time points (0, 2, 4, and 8 h) to evaluate HO-1 expression. 4-PG significantly increased HO-1 mRNA and protein levels (Figures 3(b)–3(e)). In addition, the optimal expression of HO-1 enhanced by 4-PG (10 *μ*M) was observed after 8 h treatment (Figures 3(d)–3(e)). To confirm whether 4-PG can induce HO-1 expression in human macrophages, human peripheral blood mononuclear cells (PBMC) and human monocyte cell line, U937 cells, were treated with 4-PG (10 *μ*M) at various time points (0, 2, 4, and 8 hours). We found that 4-PG significantly and time-dependently increased the expression of HO-1 in human macrophages (Figures 3(f) and 3(g)). Then, we performed a dual luciferase assay to determine the activity of the antioxidant response element (ARE), which has been identified as a major regulatory element of the HO-1 promoter, after treatment of cells with 4-PG (Figure 3(h)). 4-PG treatment significantly increased the activity of the ARE, as determined by luciferase activity. To evaluate whether 4-PG can induce other antioxidant genes, we measured the expression of antioxidant genes, thioredoxin-1 (TRX1), glutamate-cysteine ligase catalytic subunit (GCLC), and NAD(P)H dehydrogenase quinone 1 (NQO1), in RAW 264.7 cells. As expected, these antioxidant genes were significantly increased by the treatment of 4-PG (Figure 3(i)). Furthermore, we detected the anti-inflammatory effects of 4-PG on human monocytes and the lung epithelial cell line, A549 cells, respectively. 4-PG significantly decreased the mRNA expression of TNF-*α* and IL-6 in the presence of LPS (Figures 3(j)–3(l)). According to these results, we clearly demonstrated that 4-PG has the potential of increasing the expression of HO-1 in mouse and human macrophage cells and that, additionally, 4-PG exerts efficient anti-inflammatory effects on macrophages and lung epithelial cells.

### 3.4. Antioxidant Effects of 4-PG Are Mediated by HO-1 Activation

**Figure 4:**
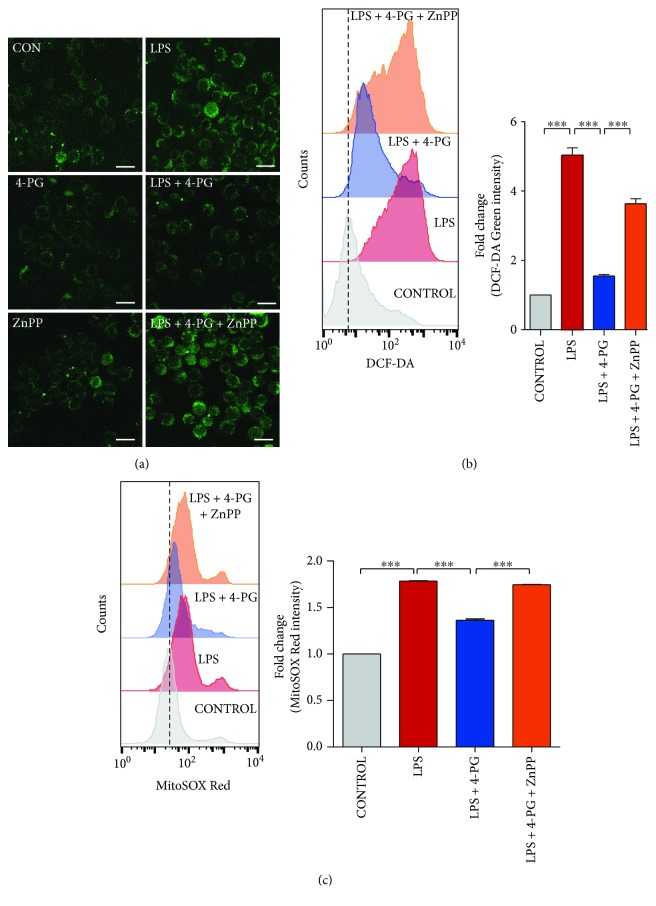
The antioxidant effect of 4-PG is mediated by HO-1 activation. (a and b) To evaluate whether the antioxidant effect of 4-PG was mediated by the activation of HO-1, RAW 264.7 cells were pretreated with the HO-1 inhibitor, ZnPP (5 *μ*M), for 30 min followed by the incubation of 4-PG (10 *μ*M) for 6 h. Then, cells were challenged with LPS (100 ng/ml) for another 4 h. The production of intracellular ROS was detected by staining DCF-DA using confocal microscopy (a) and flow cytometry (b). Scale bar = 10 *μ*m. (c) Mitochondrial ROS was stained with MitoSOX and measured by flow cytometry. Here, the fold change of fluorescence intensity is presented as mean ± SD (*n* = 5 determined in five independent experiments); ^∗∗^*p* < 0.01 and ^∗∗∗^*p* < 0.001. Comparisons were made by one-way ANOVA with Turkey post hoc tests.

In a previous study, we found that 4-PG exhibits antioxidant effects on LPS-induced oxidative stress [14]. However, the precise mechanisms by which 4-PG prevents oxidative stress remain unclear. To evaluate whether the antioxidant effect of 4-PG is mediated by HO-1, RAW 264.7 cells were pretreated with 4-PG (10 *μ*M) for 6 h followed by LPS challenge (100 ng/ml) for another 4 h. Subsequently, intracellular ROS were detected by DCF-DA staining and measured by confocal microscopy and flow cytometry, respectively (Figures 4(a) and 4(b)). We found that cells pretreated with 4-PG exhibited markedly decreased intracellular ROS production in response to LPS. Several studies have demonstrated that mtROS serve as important regulators of the inflammatory response in the innate immune system [26–28]. Based on these reports, we next tested whether 4-PG could decrease mtROS production in response to LPS. As expected, 4-PG significantly decreased the amount of mtROS (Figure 4(c)). To evaluate whether the suppression of ROS level was associated with the activation of HO-1, RAW 264.7 cells were pretreated with a HO-1 activity inhibitor, zinc protoporphyrin-IX (ZnPP). Interestingly, the antioxidant effects of 4-PG was almost completely abolished in ZnPP-treated cells during LPS challenge (Figure 4). These results suggest that LPS-induced intracellular ROS and mtROS were suppressed by pretreatment with 4-PG, the effect of which was critically mediated by HO-1 activation.

### 3.5. HO-1 Is Necessary for Antioxidant and Anti-Inflammatory Effect of 4-PG

**Figure 5:**
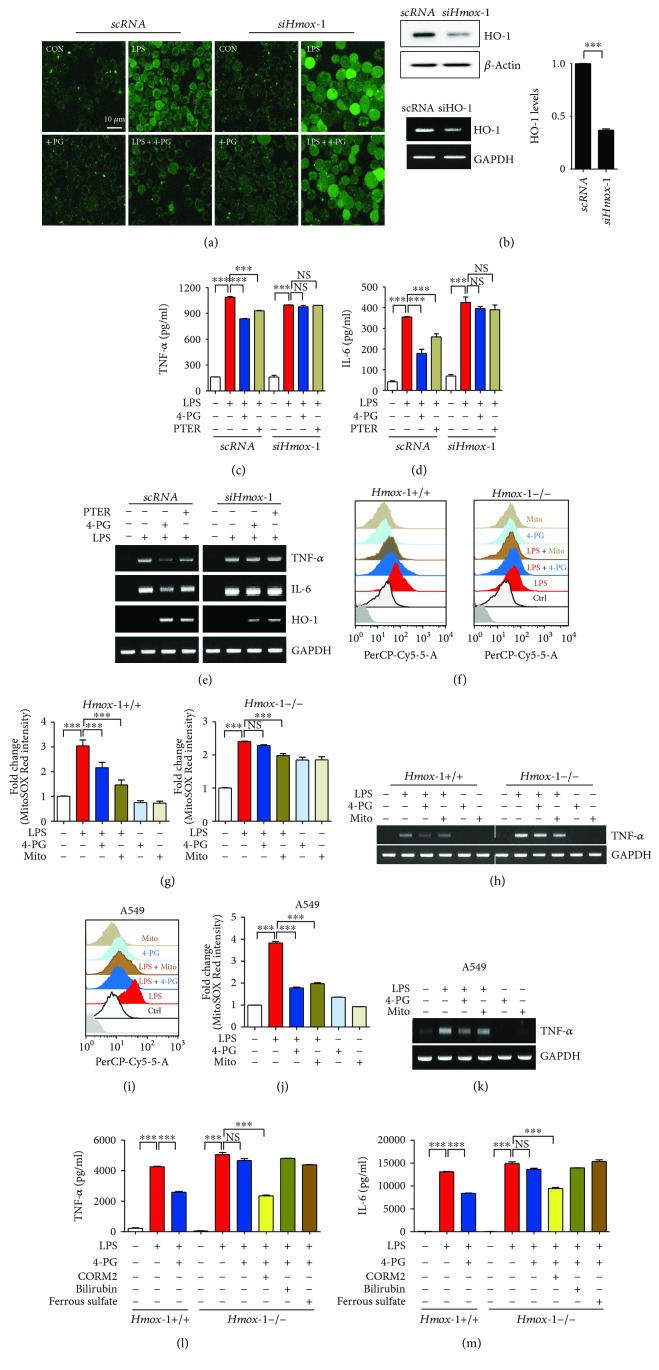
HO-1 is necessary for antioxidant and anti-inflammatory effects of 4-PG. RAW 264.7 cells were transfected with scramble RNA (*scRNA*) and siRNA against HO-1 (*siHmox-1*) for 36 h and then pretreated with either 4-PG (10 *μ*M) or PTER (10 *μ*M) for 6 h followed by stimulation of LPS (100 ng/ml) for another 4 h. (a) The production of ROS was determined by confocal microscopy. Scale bar = 10 *μ*m. (b) The mRNA and protein levels of HO-1 were determined by RT-PCR and Western blot, respectively. The conditioned supernatants were harvested, and the secreted levels of TNF-*α* (c) and IL-6 (d) in *Hmox-1^+/+^* and *Hmox-1^−/−^* BMDM were assessed by ELISA. (e) mRNA levels of HO-1, TNF-*α*, and IL-6 were determined by RT-PCR. (f–h) BMDMs, extracted from *Hmox-1^+/+^* and *Hmox-1^−/−^* mice, were pretreated with 4-PG (10 *μ*M) for 6 h and MitoTEMPO (100 *μ*M) for 30 min, respectively, and then cells were stimulated with LPS (100 ng/ml) for an additional 4 h. (i–k) A549 cells were pretreated with 4-PG (10 *μ*M) for 4 h or with MitoTEMPO (100 *μ*M) for 30 min, respectively, and then stimulated with LPS (10 *μ*g/ml) for an additional 6 h. (f and i) Mitochondrial ROS were stained with MitoSOX and measured by flow cytometry. (g and j) The fold change of fluorescence intensity is presented as mean ± SD (*n* = 5, determined in five independent experiments). (h and k) The mRNA level of TNF-*α* was determined by RT-PCR. (l and m) BMDMs isolated from *Hmox-1^+/+^* and *Hmox-1^−/−^* mice were pretreated with 4-PG (10 *μ*M) for 6 h followed by the challenge of LPS (100 ng/ml) for 4 h, and for *Hmox-1^−/−^* BMDMs, cells were copretreated with 4-PG (10 *μ*M) and HO-1 catabolites such as CO-releasing molecule- (CORM-) 2 (20 *μ*M), bilirubin (5 *μ*M), and ferrous sulfate (10 *μ*M), respectively, in the presence of LPS stimulation. Data were expressed as mean ± SD (*n* = 5 determined in five independent experiments); ^∗∗∗^*p* < 0.001. NS: not significant. Comparisons between scRNA and siHO-1-transfected groups were made by two-way ANOVA with Bonferroni post-tests.

HO-1 exerts antioxidant and anti-inflammatory effects in several diseases [29, 30]. We next evaluated whether the antioxidant and anti-inflammatory effects of 4-PG were associated with HO-1 induction. RAW 264.7 cells were first transfected with scramble RNA (*scRNA*) and siRNA targeting *Hmox-1* (*siHmox-1*). Following 36 h incubation, cells were pretreated with 4-PG (10 *μ*M) for 6 h followed by LPS (100 ng/ml) challenge for another 4 h, and then cells were stained with DCF-DA, to detect intracellular ROS by confocal microscopy. After stimulation with LPS, cells transfected with scRNA showed a significant decrease in ROS levels after administration of 4-PG, whereas the antioxidant effect of 4-PG was abolished in siHO-1-transfected cells (Figure 5(a)). Next, we investigated whether HO-1 mediated the anti-inflammatory effect of 4-PG. As shown in Figure 5(b), cells transfected with siRNA against *Hmox-1* (*siHmox-1*) exhibited significantly decreased HO-1 expression compared with cells transfected with scramble RNA (*scRNA*). In addition, we also observed that 4-PG significantly reduced both mRNA and secreted the levels of proinflammatory cytokines (TNF-*α* and IL-6) in response to LPS stimulation, which was more effective than treatment with PTER (Figures 5(c)–5(e)). However, the anti-inflammatory effects of both 4-PG and PTER were abrogated in HO-1 knockdown cells (Figures 5(c)–5(e)). Furthermore, to examine the antioxidant effects of 4-PG on primary macrophages, bone marrow-derived macrophages (BMDMs) extracted from *Hmox-1^+/+^* and *Hmox-1^−/−^* mice were pretreated with 4-PG or MitoTEMPO, a mitochondria-targeted antioxidant, and then were stimulated with LPS to detect mitochondrial ROS by flow cytometry. In BMDM isolated from wild-type mice, 4-PG and MitoTEMPO significantly decreased the mitochondrial ROS in the presence of LPS. However, compared with the *Hmox-1^+/+^* mice, the antioxidant effect of 4-PG was abolished in BMDM from *Hmox-1^−/−^* mice (Figures 5(f) and 5(g)). Moreover, to confirm whether the antioxidant effect of 4-PG was also exerted in lung epithelial cells, we pretreated A549 cells with 4-PG and MitoTEMPO during stimulation with LPS. Similar results were obtained from A549 cells, such that 4-PG and MitoTEMPO dramatically decreased mitochondrial ROS production during LPS challenge (Figures 5(i) and 5(j)). Then, we examined the anti-inflammatory effects of 4-PG on BMDM and A549 cells. 4-PG and MitoTEMPO exerted similar effects in decreasing the mRNA expression of TNF-*α* in wild-type BMDM, while in *Hmox-1^−/−^* BMDM, the anti-inflammatory effects were abrogated in the stimulation of LPS (Figure 5(h)). In A549 cells, we also observed that 4-PG and MitoTEMPO significantly decreased the mRNA expression of TNF-*α* in response to LPS. HO-1 catalyzes the rate-limiting step in the metabolic conversion of heme to biliverdin and thereby constitutes a major intracellular source of iron and carbon monoxide (CO) [16]. Biliverdin is subsequently reduced to bilirubin by the enzyme NAD(P)H biliverdin reductase [17]. Physiological concentrations of CO and biliverdin/bilirubin have been reported to exert antioxidant, anti-inflammatory, and antiapoptotic effects [17]. Heme-derived iron induces the expression of ferritin heavy chain, which mediates the cytoprotective effect of HO-1, by oxidizing the sequestered iron and thereby neutralizing the toxicity of iron [31]. Moreover, a recent study demonstrated that the induction of ferritin heavy chain (FTH) in response to polymicrobial infections was critical to establishing disease tolerance to sepsis and that the protective effect of FTH was exerted via countering iron-dependent oxidative inhibition of the liver glucose-6-phosphatase (G6Pase) to sustain endogenous glucose production via liver gluconeogenesis [32]. To identify whether the anti-inflammatory effect of 4-PG was mediated by these catabolites, bone marrow-derived macrophages (BMDMs) isolated from *Hmox-1^+/+^* or *Hmox-1^−/−^* mice were pretreated with 4-PG (10 *μ*M) followed by challenge with LPS (100 ng/ml). Additionally, *Hmox-1^−/−^* BMDMs were pretreated with 4-PG in the addition of one of three metabolic by-products of heme including CO, derived from CO-releasing molecule- (CORM-) 2 (20 *μ*M), bilirubin (5 *μ*M), or iron as ferrous sulfate (10 *μ*M), prior to stimulation with LPS (Figures 5(l) and 5(m)). Consistent with observations in RAW 264.7 cells, LPS-stimulated BMDMs displayed a significant increase in secreted proinflammatory cytokines, TNF-*α* and IL-6 (Figures 5(l) and 5(m)), while under the treatment with 4-PG, the secreted proinflammatory cytokines were remarkably decreased in *Hmox-1^+/+^* BMDMs. However, in *Hmox-1^−/−^* BMDMs, the anti-inflammatory effect of 4-PG was abolished (Figures 5(l) and 5(m)). Interestingly, we observed that CO, one of the major HO-1 catabolites, protected against LPS-induced inflammation, even in BMDMs that were derived from *Hmox-1^−/−^* mice. However, addition of bilirubin and ferrous sulfate did not exert significant beneficial effects in decreasing proinflammatory cytokines (Figures 5(f) and 5(g)). These results indicate that HO-1 plays a critical role in contributing to the antioxidant and anti-inflammatory effects of 4-PG. Moreover, CO, as one of the HO-1 catalytic products, can prevent the LPS-induced inflammatory response, and it exhibits similar anti-inflammatory effects as 4-PG. These results present that HO-1 plays a critical role in contributing to the antioxidant and anti-inflammatory effects of 4-PG. Moreover, 4-PG was more effective at attenuating the inflammatory responses than PTER.

### 3.6. HO-1 Deficiency Abolished the Anti-Inflammatory Function of 4-PG in an ALI Mouse Model

**Figure 6:**
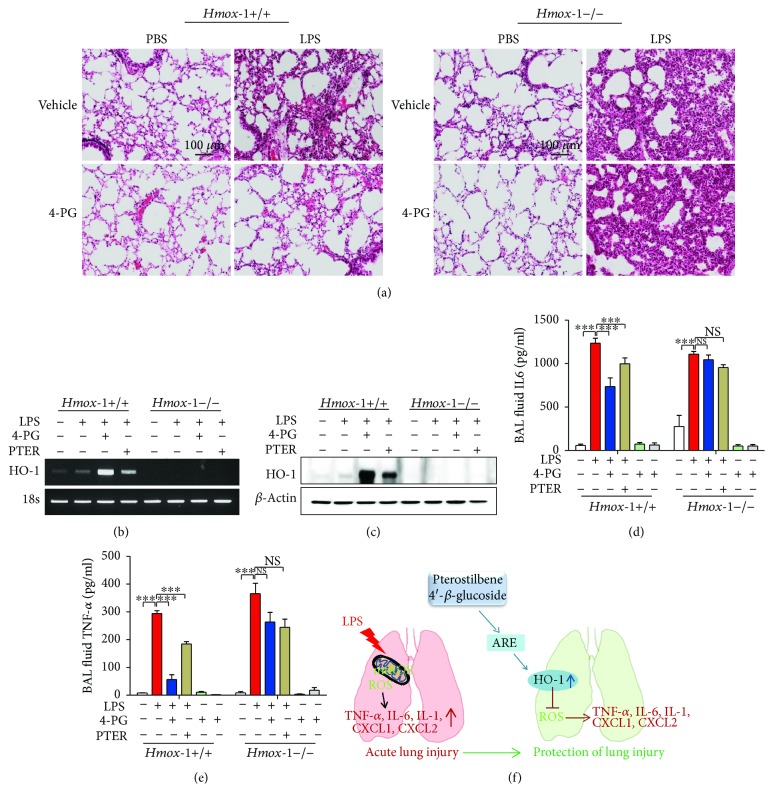
HO-1 deficiency abolished the anti-inflammatory effect of 4-PG in a mouse model of acute lung injury. 10-week-old *Hmox-1^+/+^* and *Hmox-1^−/−^* mice were injected with 4-PG (10 mg/kg, i.p.) and PTER (10 mg/kg, i.p) for 4 days prior to intranasal administration of LPS (2.5 mg/kg) for 24 h. (a) Lung sections were stained with hematoxylin and eosin (H&E) for morphological evaluation, and the representative lung sections of each group are shown. Scale bar = 100 *μ*m. The mRNA and protein levels of HO-1 from lung tissues were determined by RT-PCR (b) and Western blotting (c), respectively. BAL fluid was recovered after 24 h stimulation of LPS, and secreted levels of IL-6 (d) and TNF-*α* (e) in BAL fluid were measured by ELISA. (f) Schematic diagram of proposed pathways. 4-PG has the protective and therapeutic effects in LPS-induced ALI by increasing HO-1 expression and reducing oxidative stress. Data were expressed as mean ± SD (*n* = 5 per group); ^∗∗∗^*p* < 0.001. NS: not significant. Comparisons between genotypes at HO-1 were made by two-way ANOVA with Bonferroni post-tests.

To evaluate whether the anti-inflammatory effects of 4-PG and PTER were mediated by HO-1 in an ALI mouse model, we established an LPS-induced ALI model in *Hmox-1^+/+^* and *Hmox-1^−/−^* mice. Under normal conditions, the lung histology of *Hmox-1^−/−^* mice was comparable with that of *Hmox-1^+/+^* mice (Figure 6(a)). However, LPS administration in *Hmox-1^−/−^* mice caused much more lung damage than in *Hmox-1^+/+^* mice. 4-PG remarkably reduced lung injury in wild-type mice, but failed to protect *Hmox-1^−/−^* mice from LPS-induced lung injury. We next measured the expression of HO-1 in lung tissues of LPS-challenged mice and observed that wild-type (*Hmox-1^+/+^*) mice displayed greater expression of HO-1 mRNA and protein levels upon administration of 4-PG, than after PTER administration (Figures 6(b) and 6(c)). In BAL fluid, we measured secreted levels of the proinflammatory cytokines, TNF-*α* and IL-6. 4-PG significantly attenuated the expression of TNF-*α* and IL-6 in *Hmox-1^+/+^* mice, which was more effective than PTER. However, in *Hmox-1^−/−^* mice subjected to LPS-induced ALI, the anti-inflammatory effects of 4-PG and PTER were abolished (Figures 6(d) and 6(e)). We conclude that HO-1 plays a pivotal role in mediating the protective effects of 4-PG in the ALI mouse model and that 4-PG exhibits greater beneficial effects with respect to induction of HO-1 and the anti-inflammatory response than PTER.

## 4. Discussion

Numerous studies have demonstrated that HO-1 and its reaction products can exert antioxidant, antiapoptotic, and immune-modulatory functions in various models of cell and tissue injury [18, 33–35]. Furthermore, the HO-1-inducing compound cobalt-protoporphyrin (Co-PPIX) has been shown to significantly inhibit the expression of the proinflammatory mediators TNF-*α* and high-mobility group box 1 (HMGB1) induced by LPS in mice and thus alleviate the pathogenesis of ALI [36]. In addition, a recent study has shown that PTER, the natural dimethylated analog of resveratrol, can attenuate cerebral I/R injury by reducing mitochondrial oxidative damage via the activation of HO-1 signaling [19]. PTER has also been reported to downregulate inflammatory iNOS and COX-2 gene expression in macrophages by inhibiting the activation of NF-*κ*B [37]. In our previous study, we observed that the glycosylation product of PTER (4-PG) exerts anti-inflammatory effects in a dextran sulfate sodium-induced colitis model [14]. However, whether 4-PG can protect against endotoxin-induced acute lung injury (ALI) through the upregulation of HO-1 is still unknown.

In the present study, we demonstrated that 4-PG treatment significantly enhanced HO-1 expression in mouse lung tissues. Additionally, 4-PG exerted protective effects via the downregulation of proinflammatory cytokines and chemokines, as well as by ameliorating pathological changes in an LPS-induced ALI model in mice (Figure 1). These data suggest that both 4-PG and PTER exhibit anti-inflammatory effects in LPS-induced ALI and that 4-PG was more effective than PTER with respect to increasing HO-1 expression and decreasing proinflammatory cytokine production in lung tissue. Due to previous studies which suggested that pterostilbene has therapeutic effects in diabetes and cardiovascular diseases via the activation of Nrf2 signaling [18, 38], we sought to identify the novel therapeutic effect of 4-PG and PTER in LPS-induced ALI (Figure 2). Additionally, pneumonia represents a medical and public health priority, and advances against this disease will require improved knowledge of biological mechanisms. *P. aeruginosa* infection in the hospital manifests primarily as acute lung infection in patients in the intensive care unit (ICU). Models of pseudomonal pneumonia have highlighted the importance of classic *P. aeruginosa* virulence factors such as proteases, flagella, pili, and LPS O side chains [24]. To assess the protective effects of 4-PG on *P. aeruginosa*-induced pneumonia, in this study, in addition to the endotoxin-induced ALI animal model, we also infected mice with *P. aeruginosa* by intranasal instillation to establish a pneumonia mouse model and then post-treated with 4-PG at diverse time points (Figure 2). In mice injected with 4-PG at 5 or 6 hours after stimulation with LPS or *P. aeruginosa*, respectively, the protective effects of 4-PG were evident in significantly attenuating pathological changes of lung tissues, including the infiltration of neutrophils, as well as the expression of proinflammatory cytokines and chemokines. These results strongly demonstrate that 4-PG exerts not only preventive but also therapeutic effects on endotoxin and bacteria-induced ALI. Moreover, we also observed that early treatment of 4-PG associates with better outcomes of protection against lung injury than the late treatment. In the present study, we demonstrated the beneficial effects of 4-PG on induction of HO-1 in mouse macrophage cells as well as in human macrophage cells.

Several studies have indicated that PTER enhances HO-1 expression by increasing the nuclear translocation of Nrf2 [18, 39–41]. The PI3K-AKT signaling pathway was also shown to mediate the upregulation of HO-1 expression by resveratrol [42]. Despite our findings suggesting that 4-PG induces the expression of HO-1, the precise mechanism remains to be elucidated in the further study.

Overproduction of ROS by NADPH oxidase (NOX) isoforms has been implicated in airway and lung damage and consequently in the pathogenesis of several respiratory inflammatory diseases, including acute respiratory distress syndrome, asthma, cystic fibrosis, and chronic obstructive pulmonary disease [43]. Although ROS are generated primarily by NOX in response to stimulation with LPS, mtROS have also been proposed as important regulators of inflammatory response in the innate immune system [26–28]. The mitochondria-targeted antioxidant MitoTEMPO, which can effectively reduce the levels of mtROS, significantly decreased LPS-induced proinflammatory cytokines (TNF-*α*, IL-1*β*, and IL-6) and inflammatory mediators (iNOS and COX-2) in microglial cells [44]. PTER exhibits antioxidative effects via increasing antioxidant enzymes such as superoxide dismutase-1 (SOD1) and peroxiredoxin-4 (PRDX4) [45]. We observed that 4-PG exerts an antioxidative effect on suppressing intracellular ROS and mtROS levels, in cells which were stimulated by LPS (Figure 4). We also demonstrated, by chemical inhibition of HO activity (using ZnPPIX) or genetic interference of HO-1 using siHO-1, that the antioxidant effects of 4-PG were dependent on HO-1.

ROS can initiate inflammatory responses in the airways and lungs through the activation of redox-sensitive transcription factors, including activator protein (AP-1), hypoxia-inducible factor- (HIF-) 1, and nuclear factor-kappa-B (NF-*κ*B) [46]. In addition, overexpression of HO-1 protects against TNF-*α*-mediated airway inflammation via reduction of oxidative stress and inhibition of adhesion molecules and IL-6 expression in both cultured human tracheal smooth cells and the airways of mice [47]. Based on these reports, we evaluated whether HO-1 mediates anti-inflammatory effects of 4-PG *in vitro* and *in vivo*. We found that 4-PG significantly decreased LPS-induced proinflammatory cytokines (TNF-*α* and IL-6) and mRNA and protein levels. However, in HO-1 knockdown cells or *Hmox-1*-deleted mice, the protective and therapeutic effects of 4-PG were abolished, and the pathological changes in lung tissues were more severe in response to LPS challenge. Moreover, we also observed that 4-PG was more effective than PTER at downregulating TNF-*α* and IL-6. In light of these results, we conclude that 4-PG exerts anti-inflammatory effects, which are primarily mediated by HO-1. It is well known that HO-1, a key metabolic enzyme, catalyzes the degradation of heme and generates biologically active reaction by-products including biliverdin, ferrous iron, and carbon monoxide (CO) [16]. To identify which catabolites of HO-1 play an important role in attenuating the inflammatory responses induced by LPS, we pretreated BMDMs derived from *HO-1^−/−^* mice with bilirubin, ferrous sulfate, or CORM2, in the presence of 4-PG. Following stimulation with LPS, we found that only CO (as CORM2) exerts an influence on reducing proinflammatory cytokines. However, the other two catabolites of HO-1, iron (as ferrous sulfate) and bilirubin, exerted slight but not significant anti-inflammatory effects during LPS challenge. These results suggest that CO, generated from HO-1, may mediate the anti-inflammatory effect of 4-PG. In a previous study, we have demonstrated that tristetraprolin (TTP) plays a crucial role in mediating the protective effect of 4-PG and CO in DSS-induced colitis [14, 35].

HO-1 and TTP have also been shown to be functionally linked in mediating the anti-inflammatory function of nicotine. Nicotine enhances the expression of HO-1, and HO-1-dependent STAT3 signaling increases TTP levels, which in turn inhibits LPS-induced TNF-*α* production [48]. According to these reports, further studies will be required to clarify whether 4-PG-induced TTP expression is regulated by the HO-1/STAT3 signaling pathway.

In conclusion, our results demonstrate for the first time that the antioxidant and anti-inflammatory effects of 4-PG are mediated by the activation of HO-1 and that 4-PG is more effective than PTER in conferring anti-inflammatory protection. Additionally, *Hmox-1* deficiency abolished the protective and therapeutic effects of 4-PG against lung pathologic changes in LPS- or *P. aeruginosa*-induced ALI. These findings establish that 4-PG can exert critical protective effects in ALI via HO-1 activation and provide an avenue for therapeutic intervention of respiratory inflammatory diseases.

## Data Availability

The data used to support the findings of this study are included within the article.

## Abbreviations

4-PG: Pterostilbene 4′-*β*-glucoside
ALI: Acute lung injury
BAL: Bronchoalveolar lavage
HO-1: Heme oxygenase-1
IL-6: Interleukin-6
LPS: Lipopolysaccharide
mtROS: Mitochondrial reactive oxygen species
NF-*κ*B: Nuclear factor-kappa B
PTER: Pterostilbene
ROS: Reactive oxygen species
TNF-*α*: Tumor necrosis factor-alpha
TTP: Tristetraprolin.
